# Feasibility Evaluation of Metamaterial Microwave Sensors for Non-Invasive Blood Glucose Monitoring

**DOI:** 10.3390/s21206871

**Published:** 2021-10-16

**Authors:** Lukas Malena, Ondrej Fiser, Paul R. Stauffer, Tomas Drizdal, Jan Vrba, David Vrba

**Affiliations:** 1 Faculty of Biomedical Engineering, Czech Technical University in Prague, 160 00 Prague, Czech Republic; lukas.malena@fbmi.cvut.cz (L.M.); ondrej.fiser@fbmi.cvut.cz (O.F.); tomas.drizdal@fbmi.cvut.cz (T.D.); jan.vrba@fbmi.cvut.cz (J.V.); 2 Department of Radiation Oncology, Thomas Jefferson University, Philadelphia, PA 19107, USA; Paul.Stauffer@jefferson.edu

**Keywords:** dielectric properties, glucose monitoring, microwave sensor

## Abstract

The use of microwave technology is currently under investigation for non-invasive estimation of glycemia in patients with diabetes. Due to their construction, metamaterial (MTM)-based sensors have the potential to provide higher sensitivity of the phase shift of the *S*_21_ parameter ($∠S_{21}$) to changes in glucose concentration compared to standard microstrip transmission line (MSTL)-based sensors. In this study, a MSTL sensor and three MTM sensors with 5, 7, and 9 MTM unit cells are exposed to liquid phantoms with different dielectric properties mimicking a change in blood glucose concentration from 0 to 14 mmol/L. Numerical models were created for the individual experiments, and the calculated S-parameters show good agreement with experimental results, expressed by the maximum relative error of 8.89% and 0.96% at a frequency of 1.99 GHz for MSTL and MTM sensor with nine unit cells, respectively. MTM sensors with an increasing number of cells show higher sensitivity of 0.62° per mmol/L and unit cell to blood glucose concentration as measured by changes in $∠S_{21}$. In accordance with the numerical simulations, the MTM sensor with nine unit cells showed the highest sensitivity of the sensors proposed by us, with an average of 3.66° per mmol/L at a frequency of 1.99 GHz, compared to only 0.48° per mmol/L for the MSTL sensor. The multi-cell MTM sensor has the potential to proceed with evaluation of human blood samples.

## 1. Introduction

Blood glucose level is an important diagnostic indicator. Glucose monitoring is most often performed in the case of diabetes, which is one of the most common chronic diseases. Diabetes is manifested by insufficient production or utilization of the hormone insulin, which is produced by the ß-cells of the pancreas [1]. The cause of diabetes is mainly unhealthy lifestyle and genetic factors. The total number of cases exceeds 400 million, and over 1 million diabetics die each year. In 2021, up to 10% of the population in developed countries is at risk of diabetes, and the trend is deteriorating due to ageing [2,3].

Diabetes is manifested by a disorder of carbohydrate metabolism and insulin resistance. The consequence of the disease is fluctuations in blood glucose values outside the range of 3.9–5.6 mmol/L, which leads to two serious conditions. Hypoglycemia is a diabetes condition in which the plasma glucose level in an adult diabetic falls below 3 mmol/L, which in some unfortunate cases leads to hypoglycemic coma and death [1]. The risk of hypoglycemia during sleep leads to the need to measure at shorter intervals, which can cause patients a high degree of discomfort. The reverse case is hyperglycemia, where the blood glucose level exceeds 7 mmol/L when fasting. If this condition persists chronically, it is most often manifested by damage to blood vessels in the retina of the eye and kidneys, or damage to sensory nerves. In some cases, a patient’s body suffering from acute insulin deficiency is brought into a state of diabetic ketoacidosis in an effort to quickly gain energy by breaking down fats. Ketoacidosis can again lead to coma and possible collapse of the body. The above-mentioned manifestations lead to a need for acute intervention by a medical doctor or require long-term hospitalization [1]. An indicator of properly set treatment is the blood glucose level [1,4]. Due to the many specific properties of glucose, which is needed for each cell in the body, several types of assays without the need to penetrate the vasculature invasively are currently being developed [5].

Non-invasive glucose sensors can be based on the detection of glucose in body fluids, which uses the relationship between the concentration of glucose in a given body fluid and blood. Other detection principles are based on bioimpedance, infrared and Raman spectroscopy, optical coherence tomography, reverse iontophoresis, fluorescence techniques, or ultrasound and microwave technology [6,7,8]. Some devices are already commercially available for patients with Type II diabetes, for which accurate blood glucose values are not required for diagnosis. The GlucoTrack system from Integrity Applications uses a combination of the above-mentioned types of sensors located in a clip that attaches to the earlobe [9]. The system from Cnoga Medical works on the principle of NIR spectroscopy [10]. When using the system, it is necessary to calibrate by the classical invasive method, which needs to be repeated over time. The most recent device launched on the market is the GlucoBeam instrument based on the principle of Raman spectrometry [11,12]. This device is relatively robust, but the authors now face the challenge of modifying it to allow continuous measurement with improved patient comfort [6].

Research into the mechanisms by which glucose in a patient’s blood affects the electromagnetic field has the potential to provide a new and rapid way to measure blood glucose non-invasively. One promising approach is the use of microwaves that can penetrate biological tissue to a depth of several millimeters, or more if desired [13]. Microwave sensors are based on the principle of interaction of microwave radiation with the biological tissue through which it passes [14,15]. Radiation through tissue is significantly affected by the tissue’s electrical properties, including electrical conductivity and dielectric constant. Since the amount of glucose and its biological activity directly affects the dielectric properties of blood, we can use microwave sensors to determine the glycemic value. However, the changes in dielectric properties are very small, which is why scientific teams are focusing on increasing the sensitivity of the proposed sensors [16,17,18,19].

A microwave sensor for measuring the blood glucose level is usually designed as a resonator with planar geometry to couple across the skin surface. In most cases, microwave blood glucose sensors are designed to change their resonant frequency in direct proportion to changes in tissue dielectric parameters. Some investigators use a patch antenna or various types of microstrip lines for glucose detection [20,21,22,23]. Unlike resonance-based sensors, antennas can alternatively be based on the principle of modulating the amplitude and phase of S-parameters. Some have already been used for in vivo measurements and have been shown to be able to estimate glycemic concentrations in real time. At this time, microwave sensors require individual calibration for each subject with an invasive glucometer [20,24,25,26,27,28].

The proposed microwave sensor which was first introduced and described in [29] uses a section of planar metamaterial transmission line (TL) operating at 2 GHz. At this frequency, the ∠*S*_21_ parameter is highly affected by blood glucose level. Due to the unique sensor design and measurement approach, a higher sensitivity of sensor response in a lossy dielectric environment is expected.

The main goal of this paper is to validate the feasibility of a high-sensitivity planar metamaterial TL-based blood glucose sensor. For this purpose, a prototype sensor will be fabricated and used to measure ∠*S*_21_ of liquid phantoms mimicking the expected range of blood glucose levels, and checked against numerical simulations.

## 2. Materials and Methods

### 2.1. Sensor Design

In general, transmission-line-based sensors evaluate the phase change of the transmission coefficient, caused by a change in the dielectric (permittivity and conductivity) and magnetic properties of the environment in their immediate vicinity. Since most biological substances can be considered as non-magnetic, only changes in dielectric properties are evaluated in the considered application. The sensitivity of the sensor to changes in the dielectric parameters of the environment is directly related to changes in the phase constant. The main circuit element, the value of which affects the phase constant, and which is directly dependent on the dielectric parameters, is the capacitor.

**Figure 1:**
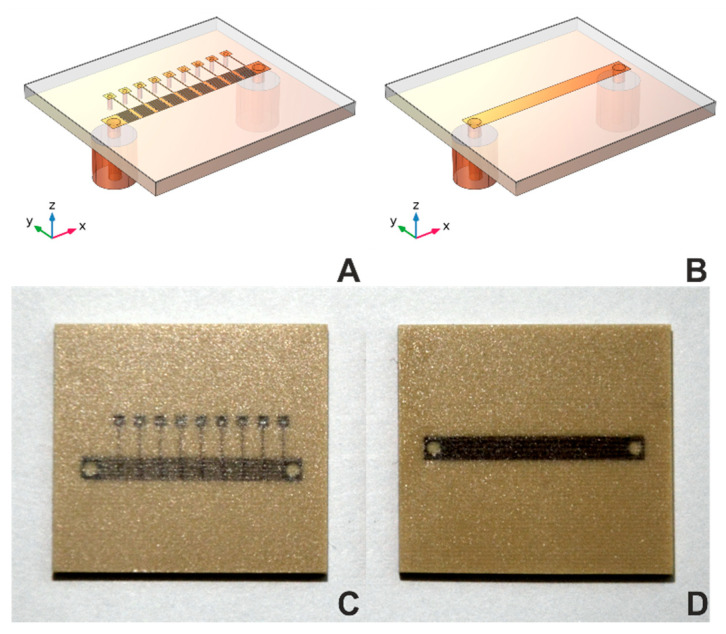
Geometries and photographs of sensors. Geometries of (**A**) MTM9 and (**B**) MSTL sensors. Photographs of (**C**) MTM9 and (**D**) MSTL sensor.

In the equivalent MSTL electrical circuit, there is only a parallel capacitance C_p_ [30]. Its value is sensitive to changes in the dielectric properties of the matter under test (MUT) only due to the fringing electric fields partially entering the MUT. The contemplated MTM structure also has C_p_. Unlike the MSTL, the main sensing elements here are intentionally introduced longitudinal capacitors C_s_ (implemented as interdigitated capacitors in Figure 1A,C), located as close as possible to the MUT [31].

Our current effort introduces a metamaterial transmission line (MTM) sensor concept. Each sensor consists of N unit cells, which includes serial interdigital capacitors and shunt inductor, collectively forming a composite right/left-handed (CRLH) structure, as described in [29] (see Figure 1A,C). For comparison of sensor sensitivity, we chose MTM sensors with five, seven, or nine-unit cells vs. a standard MSTL sensor (see Figure 1B,D). All sensors were microwave 2-port devices with the same length, substrate, and coating layer, and were mounted into a 30 mL container [32]. Corresponding numerical models of transmission line MSTL and the nine-cell metamaterial (MTM9) were created in software COMSOL Multiphysics. For sensitivity evaluation, we used the relation between phase shift Δ$∠S_{21}$ of transmission coefficient *S*_21_ and equivalent blood glucose concentration, given by the analytical model *c*_bg_, defined as:

(1) $$\phi =\frac{\Delta ∠S_{21}}{\Delta c_{bg}},$$

where ϕ is sensor sensitivity in mmol/L.

### 2.2. Phantom for Sensors Testing

Using human blood for initial experiments with the microwave blood glucose sensor brings many requirements for biological hazard risk management and blood sample processing. The microwave blood glucose sensor detects changes in relative permittivity and electrical conductivity of a sample. To facilitate initial investigations, blood may be substituted by a tissue-mimicking substance with the same dielectric parameters, also known as a phantom. In this study, we use a mixture of isopropyl alcohol (IPA), deionized (DI) water, and sodium chloride for mimicking dielectric parameters of blood with a dissolved glucose range from 0 to 14 mmol/L [33]. The exothermic reaction of isopropyl alcohol with deionized water should be mentioned because dielectric properties are also affected by sample temperature. The raw mixture of the aforementioned components is then placed in an ultrasound bath for three minutes for degassing and homogenization. According to the mathematical model from [34] we calculated the values of blood dielectric parameters for various blood glucose concentrations shown in Table 1. Across this typical range of blood glucose concentrations and corresponding dielectric properties, the required sensor measurement sensitivity remains unchanged: to detect changes of one unit in relative permittivity and 0.1 S/m in electrical conductivity.

### 2.3. Dielectric Parameters Measurement

The measurement of dielectric properties was conducted using the dielectric assessment kit (DAK12) coaxial probe, swept across the frequency range 1.5–2.5 GHz in 10 kHz steps [35]. Due to the DAK12 probe diameter of 48 mm, we used a 500 mL sample volume. Rough electrical conductivity adjustments were made by adding sodium chloride to achieve 0.1 S/m lower than the desired values. Decreasing the sample temperature and dissolving sodium chloride in the mixture affects the final electrical conductivity values. After thorough stirring and ultrasound homogenization, the samples were cooled to room temperature of 25 °C. Dielectric properties of the phantom mixtures were then adjusted to their final desired values at a center frequency of 2 GHz by adding small volumes of deionized water to increase relative permittivity, or isopropyl alcohol, which has an inverse effect and also increases electrical conductivity. For all sensors, 10 independent measurements were performed on 2 mL and 4 mL samples of each phantom. These volumes correspond to standard blood sample tubes.

### 2.4. S-parameter Measurements

**Figure 2:**
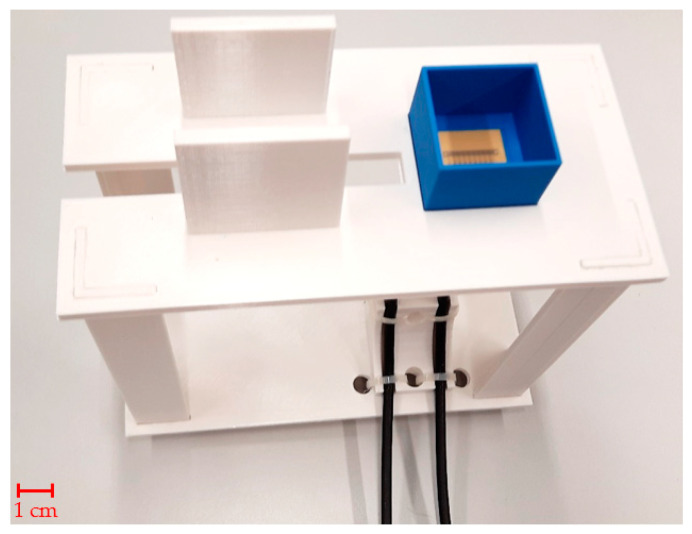
Tailor-made holder with MTM9 sensor in PLA box ready for measurement.

S-parameters are electric circuit parameters (similar to impedance, admittance, or H- parameters) used mainly in high-frequency and microwave technology. They describe the electrical behavior of the circuits by the ratios of incident, reflected, and complex magnitudes of voltage waves on individual ports [30]. The measurement of sensor S-parameters was performed by vector network analyzer (VNA) Rohde & Schwarz ZNB 8 [36] with 1 MHz resolution between 1.5 and 2.5 GHz. The power was set to a maximum value of 13 dBm. A Rohde & Schwarz ZN-Z152 calibration unit was used to calibrate the vector network analyzer with reference planes set at the sensor’s ports. The calibration unit was placed onto a custom 3D-printed holder, depicted in Figure 2, which facilitates easy connection of test sensors without displacing the coaxial cable setup. Using a pipette, the measurement chamber was filled with 2 mL of phantom 10 times, and S-parameters were measured 10 times and stored in a Touchstone file for subsequent import to MATLAB. The 10 independent measurement procedures were repeated with 4 mL phantom samples.

### 2.5. Numerical Simulations

Numerical simulations were performed for the MSTL sensor with a strip width of 2.1 mm and for the MTM sensor with 9 unit cells whose geometry is taken from [29]. Numerical models of the sensors were created in the well-proven commercial numerical simulator COMSOL Multiphysics. Full-wave electromagnetic field simulations in the frequency range from 1.859 to 2.5 GHz were performed for both sensors for five glucose concentration values. Subsequently, the S-parameters were calculated using a workstation equipped with two INTEL XEON silver 4208 processors and 192 GB of RAM.

#### 2.5.1. Computational Domain Geometry

**Figure 3:**
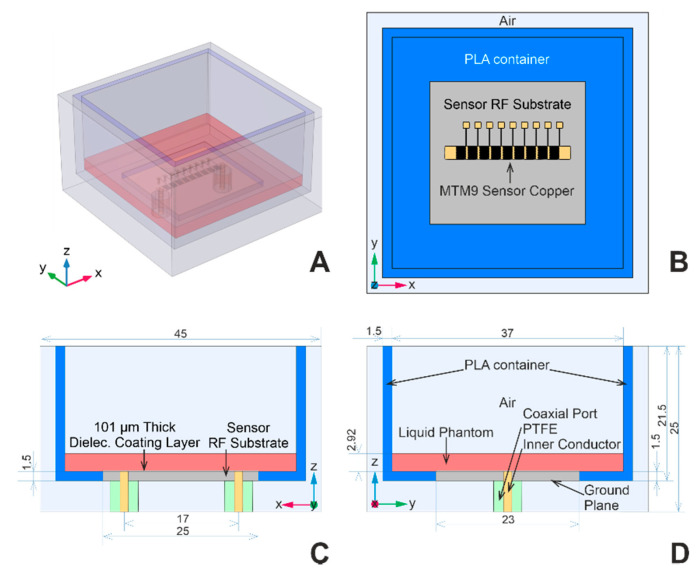
Computational domain geometry; (**A**) and (**B**) are perspective and top view of the computational domain geometry, respectively. (**C**) and (**D**) are detailed views of the cross-sections in xz and yz planes with dimensions in mm.

The computational domain geometry includes one of the sensors—MTM9 and MSTL, whose geometries are described in detail in [22]. For readers’ convenience, partially transparent models revealing the internal structures of MTM9 and MSTL are depicted in Figure 1A and 1B, respectively. Each sensor within the numerical model is virtually placed in the blue polylactide (PLA) container, filled with the liquid phantom represented by red block, and surrounded by air (see Figure 3A). Rogers Ro4003c substrate was coated with 101 μm-thick layer of MT40 laminate. The RF substrate is shown in Figure 3B–D, rendered in gray color. Underneath the RF substrate is located a copper ground plane connected with two sections of coaxial lines representing SMA ports.

#### 2.5.2. Dielectric Properties of Computational Domains

The dielectric parameters of the individual computational domains are given in Table 2. The value of relative permeability of all domains is equal to 1. Frequency dependencies of relative permittivity and electrical conductivity of individual samples measured with the DAK system were approximated in accordance with the determination coefficient by polynomial Equation (2) [37]:

(2) $$\varepsilon_{r}\left(f\right)=p_{3}f^{3}+p_{2}f^{2}+p_{1}f+p_{0},$$

and Equation (3):

(3) $$\sigma_{e}\left(f\right)=q_{1}f+q_{0},$$

respectively, and implemented in numerical models. The corresponding polynomial functions’ coefficients *p_n_* and *q_n_* are listed in Table 3.

#### 2.5.3. Discretization Mesh Settings

The discretization mesh was set in accordance with the simulator manufacturer’s recommendation—the maximum edge length of the discretization tetrahedrons is less than one-fifth of the minimum wavelength of a plane electromagnetic wave in the given material and the considered frequency range. The mesh density on the surfaces representing the copper motifs of the sensors and in the domains representing the coating layer was intentionally increased to get a higher precision of the simulation results.

First, the surfaces representing the copper motif of the sensors were discretized. In the case of the MSTL sensor, the mesh parameters were set so that the mesh contained at least six layers of triangular elements along the width of the microstrip. In this way, it was possible to accurately simulate the high current density along the outer edges of the MSTL. For the MTM sensor, the maximum edge length of the triangular mesh was set at 0.035 mm due to the small width of the fingers and the gaps between the fingers of the interdigitated capacitors, which are 0.1 mm. The mesh density of the coating layer was increased in the *z*-direction by a factor of 4 to include at least two layers of tetrahedrons per domain thickness.

After an initial evaluation of the results of the numerical simulations, the density of the triangular mesh of the copper motif representing the surfaces was increased so that the number of network elements increased by 50% of the original value. The resulting maximum lengths of the edges of triangles and tetrahedrons in the individual computational domains of the model are given in Table 2.

#### 2.5.4. Additional Settings of Numerical Models

The outer surface of the computational domain was provided with a “scattering boundary condition”, which reduced the calculated reflections of electromagnetic waves incident on the outer surface of the computational domain. The only exception was areas representing coaxial ports, which were provided with the corresponding port boundary conditions.

The copper parts of the sensors were simulated using two types of boundary conditions. First we use the “Perfect Electric Conductor” (PEC) boundary condition, which is usually worth considering for the selected frequency band and for lossy structures, as well as biological tissues. The second considered “transition boundary condition” takes into account electrical losses in copper and its surface irregularities [41].

### 2.6. Data Evaluation

Each liquid phantom was measured ten times with the DAK system, and the results were assigned a type c measurement uncertainty.

In addition, the measurement of the response of sensors to liquid phantoms was performed ten times using vector network analyzer ZNB-8, Rohde & Schwarz, Germany. The ∠*S*_21_ was unwrapped and assigned a type c measurement uncertainty. The data were further analyzed for individual frequency points, where for each frequency point the decreasing order of the samples was checked and a linear regression model of the dependence of the change $∠S_{21}$ on the theoretical glucose concentration was prepared.

Root mean square error (RMSE) was calculated for all sensor types from linear regression model data and measured values. According to the value of the RMSE, a frequency showing a higher degree of linear dependence was selected. Furthermore, the RMSE calculation was used to evaluate the effect of changing the settings of numerical simulations. In this case, we monitored the RMSE value between measured and simulated $∠S_{21}$. We used Equation (4) for the RMSE calculation [42]:

(4) $$RMSE=\sqrt{\frac{1}{n}\overset{n}{\underset{i=1}{\sum }}{\left(\hat{\theta }-\theta \right)}^{2}},$$

where $\hat{\theta }$ and θ are the simulated and measured value, respectively, and n is the number of samples.

## 3. Results

### 3.1. Liquid Phantoms

A total of five liquid phantoms were prepared by mixing deionized water, isopropyl alcohol, and sodium chloride according to the composition in Table 4.

### 3.2. Dielectric Properties of Phantoms

**Figure 4:**
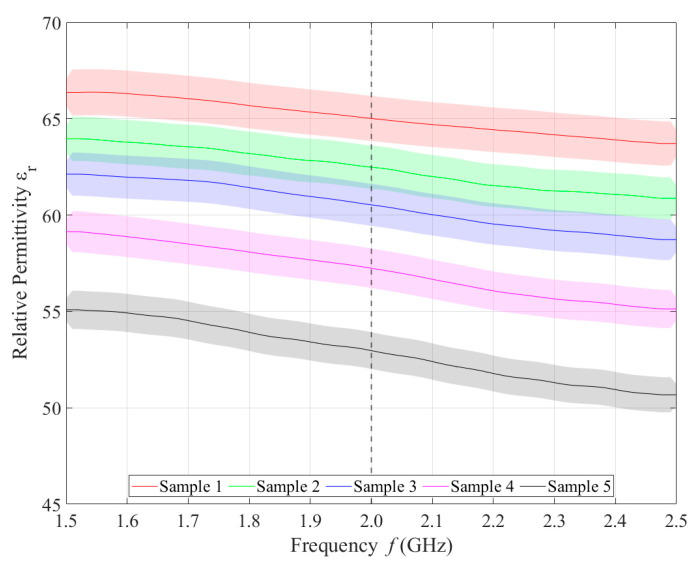
Measured values of relative permittivity.

**Figure 5:**
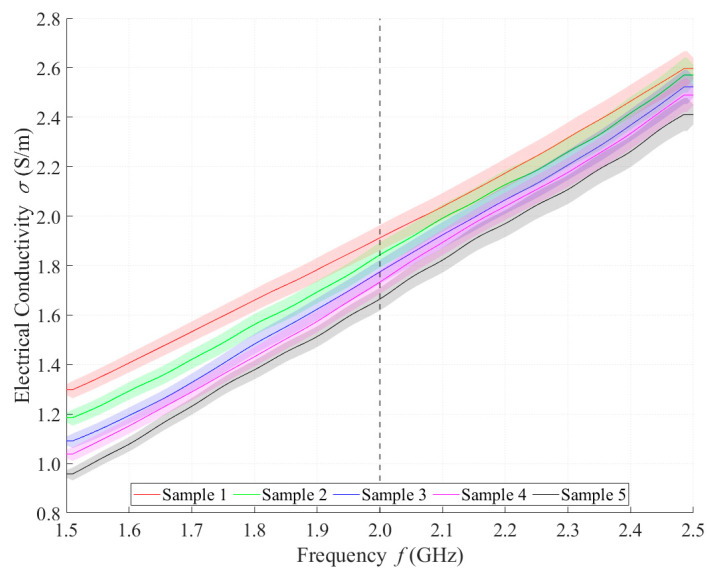
Measured values of electrical conductivity.

Relative permittivity (Figure 4) and electrical conductivity (Figure 5) of liquid phantoms were measured at room temperature (25 °C) using a DAK12 system. Because the sensors were designed for an operating frequency of 2 GHz, the values of the phantoms’ dielectric properties were set for this particular frequency and are shown in Table 5. Each sample is marked in the graph with a specific color that will be used in all subsequent graphs. Furthermore, the corresponding measurement uncertainty of type c is also indicated for each sample by the color band.

### 3.3. VNA Measurement

**Figure 6:**
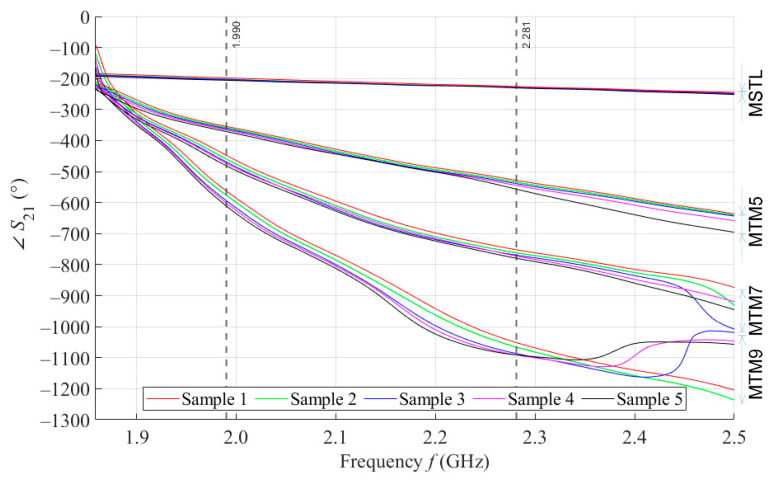
The course of ∠*S*_21_ for all tested sensors.

By performing a broadband (1.5–2.5 GHz) VNA measurement, we want to examine the behavior of our proposed sensors in wide surroundings of the designed operating frequency of 2 GHz. Figure 6 shows the courses of $∠S_{21}$ parameter values for 5 samples with a volume of 4 mL measured by MSTL and three types of MTM sensors with 5, 7, and 9 cells. The values are, for better clarity, depicted without c type measurement uncertainty. As the number of unit cells of the MTM sensor increases, the frequency at which the sensor responds by changing of the $∠S_{21}$ parameter increases, too. For this reason, an inflection point was found for the course of the $∠S_{21}$ parameter of the MTM9 using the second derivative. The inflection point determines the frequency of 1.859 GHz, from which all sensors respond to phantoms by changing of $∠S_{21}$ parameter.

We further assume that for future experiments, only one operating frequency will be needed. In order to ensure the stability of the measurement, the final operating frequency of 1.99 GHz was chosen. At this frequency, the $∠S_{21}$ parameters of all sensors were sorted in descending order. In addition, in the vicinity of the final operating frequency 1.99 GHz, the RMSE values between the theoretical linear model of glucose concentration and the $∠S_{21}$ measured values calculated according to Equation (4) reach a local minimum. The highest frequency at which all proposed sensors provide us courses of $∠S_{21}$ arranged in descending order determines the last functional frequency of 2.281 GHz. Above this value, there is not monotonic response of $∠S_{21}$ parameters for MTM9, and at higher frequencies also for MTM 7. Furthermore, the increasing sensitivity of MTM sensors with the increasing number of cells calculated according to Equation (1) was confirmed by numerical simulation. The average sensitivity of the $∠S_{21}$ parameter in the measured range of glucose concentrations is given in Table 6.

### 3.4. Measurement vs. Simulation

**Figure 7:**
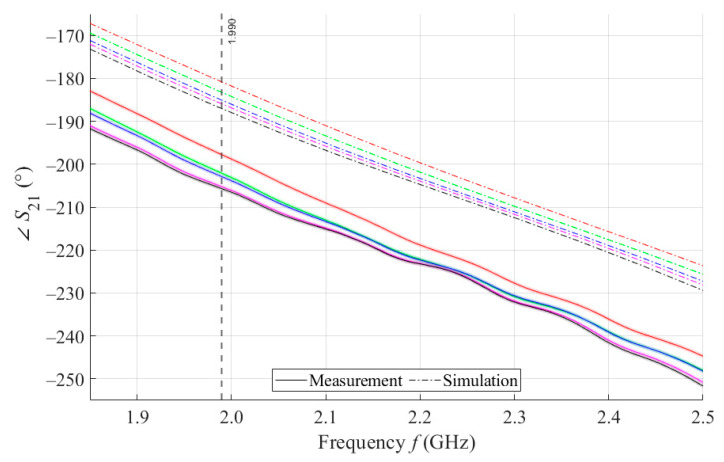
The course of ∠*S*_21_ for MSTL and 4 mL.

The results of measurements and simulations for MSTL and MTM9 sensors show agreement in trends of the amplitude and phase of *S*_11_ and *S*_21_ parameters. The main mo-nitored variable in this work is the phase of parameter *S*_21_, whose waveforms for individual samples for both measurements and simulations are shown in Figure 7.

**Figure 8:**
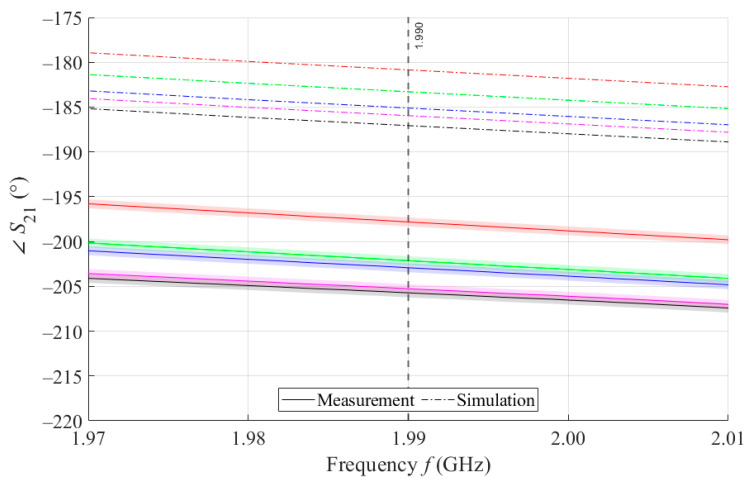
Detail of MSTL at 1.99 GHz and 4 mL.

**Figure 9:**
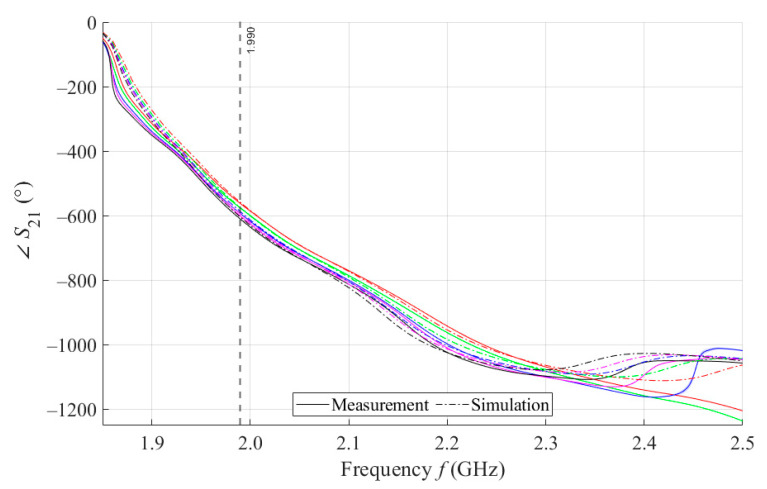
The course of ∠*S*_21_ for MTM9 and 4 mL.

**Figure 10:**
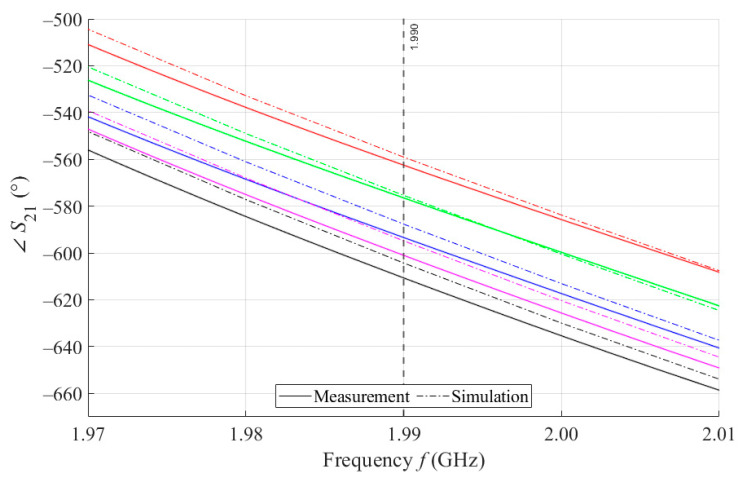
Detail of MTM9 at 1.99 GHz and 4 mL.

As can be seen in Figure 8, which is a detail of Figure 7, the simulation for MSTL at 1.99 GHz showed a phase deviation of 17.8° on average compared to the measurements. This phase difference can be observed over the entire frequency bandwidth. The course of the $∠S_{21}$ parameter for MTM9 is depicted in the Figure 9. In contrast, for MTM9, the average absolute deviation between the measured and simulated phases at the same frequency is only 5.48° (see Figure 10). Thus, for MTM9 a good agreement was achieved between the numerical simulation and the measurement itself of RMSE = 0.76 at the frequency of 1.99 GHz. For comparison between measurements and numerical simulations, the average sensitivity of the $∠S_{21}$ parameter for MSTL and MTM9 sensors was also evaluated for synthetic data (see Table 6). Values of $∠S_{21}$ for MSTL and MTM9 obtained by measurement are provided with a confidence interval showing the uncertainty of type c. It should be noted that in Figure 9 and Figure 10, it is necessary to show a larger range of $∠S_{21}$ due to the higher sensitivity of MTM9. The average value of type c uncertainty for MTM 9 is ±0.36°, if we keep the same frequency range in Figure 9 and Figure 10 as in Figure 7 and Figure 8, the confidence intervals appear very narrow.

The power balance and electromagnetic energy losses in individual parts of the system were investigated. Most energy was lost in the phantom of the blood (92.5%), in the coating layer (5.1%), and in the dielectric substrate (2.4%).

## 4. Discussion

### 4.1. Microwave Sensors

The printed circuit boards of the microwave sensors were manufactured by a professional circuit board manufacturer based on a digital design. Each sensor consists of a Rogers RO4003 substrate for high-frequency applications, which is coated with a layer of high-frequency laminate Isola I-Terra MT40 and connection ports provided via SMA coaxial connectors. A tailor-made holder proved valuable, enabling quick replacement of sensors without changing the position of the measuring cables connected directly to the VNA. The future experiments will also aim to find the optimal position of proposed sensor on skin surface. The final region where the future device will be used should have sufficient blood perfusion. The sensor could be, for example, in a handheld device placed against the carotid artery, in a “watch band”, or in a finger or ear lobe clip.

### 4.2. Liquid Phantoms for Sensor Testing

The preparation of phantoms requires precise analytical work using chemical laboratory equipment. We used calibrated analytical balances to weigh the individual components and volumetric flasks and calibrated automatic pipettes to determine the volumes. The deionized water comes from a professional laboratory apparatus with monitored electrical conductivity. We used analytical grade sodium chloride and purified isopropyl alcohol to achieve repeatable electrical conductivity and relative permittivity. Dissolving sodium chloride in solutions with a higher isopropyl alcohol content requires good mixing. For this reason, we used an ultrasonic bath, which also removes gas bubbles, which otherwise might adhere to the DAK coaxial probe and distort the dielectric parameter measurements. In the ultrasonic bath, the phantom solution is heated during stirring, and therefore it is necessary to continue measurements with the DAK system until the solutions have cooled to room temperature. In our case, we let the phantom mixtures stand in a closed storage bottle until they achieved a temperature of 25 °C. It should be noted that the isopropyl alcohol affects both relative permittivity and electrical conductivity. In addition, between the samples there is a relatively small change in dielectric properties, making it relatively difficult to obtain a series of solutions with exact concentration values, as in the mathematical model.

The phantom properties could be affected by the quality of the input ingredients and the precision of the workflow. To ensure the stability of the measurement, the DAK system itself was put into operation at least one hour before the final calibration and the actual measurement of the dielectric properties of the liquid phantoms. All five samples were measured in a vessel of the same shape ten times independently. The difference between the theoretically calculated and measured values in most cases fit into the confidence interval given by the uncertainty of type c. At a frequency of 1.99 GHz, the absolute error of relative permittivity and electrical conductivity reached a maximum of 0.62 and 0.04 S/m, respectively. Because the same liquid phantoms were used to test all types of sensors, there was no need to recalculate glucose concentrations on a new scale to account for variations in liquid phantom manufacturing.

In addition, it must be taken into account that the available models of the dependence of the dielectric parameters of blood on glucose content differ in the case of relative permittivity by up to several units. In the initial phase of design and testing of MTM sensors, our primary focus was to demonstrate the ability to detect changes in the dielectric properties of liquid phantom. In the next phase of research, liquid phantoms will be replaced by blood samples with known glycemia and dielectric parameters. At that point in the research, it will be possible to link the results of MTM sensor $∠S_{21}$ measurements to specific glycemic values indicated by the validated assay and to expand knowledge about practically achievable accuracy and resolution of the proposed MTM sensor [15,34,43].

### 4.3. VNA Measurements Assessment

Measurement accuracy is limited by the minimum sample size for all sensors. As described in Section 2.3, two sets of 10 independent measurements were performed for all sensors with a sample volume of 2 mL and 4 mL. These two volumes were selected to maintain compatibility with the standard blood collection tubes that we intend to use in future work. The courses of $∠S_{21}$ for five samples with a volume of 2 mL, which corresponds to one standard sample tube, were not sorted in descending order. The cause of this phenomenon was the high variance of the $∠S_{21}$ between the 10 independent measurements observed with all types of sensors, which could be caused by adhesive forces between the surface of the PLA box, sensor, and liquid phantom. The results showed that for accurate measurements, the minimum required volume must be 4 mL.

From the course of $∠S_{21}$ parameters in Figure 6, it is clear that MTM sensors show a higher sensitivity than the MSTL. Furthermore, the $\Delta ∠S_{21}$ between samples increases with number of cells of MTM sensors, as seen in the increasing average sensitivity values in Table 6. The advantage of the MTM sensor is also high sensitivity in the hypoglycemic area. Another observed phenomenon associated with the number of MTM sensor cells is the gradual narrowing of the available measurement frequency band, in which there is a monotonic response of $∠S_{21}$ parameters. As already described in Section 3.3, only the final operating frequency of 1.99 GHz could be considered for future work. If we will want to change the operating frequency while maintaining the current MTM9 sensor design, we are limited by the frequency band, starting at the frequency of 1.859 GHz, from which the sensor begins to respond by changing $∠S_{21}$, up to the frequency of 2.281 GHz, where the courses of $∠S_{21}$ cross each other. There could be also an option of redesigning the sensor for a higher operating frequency, but the sensor is intentionally designed at a relatively low operating frequency of 2 GHz so that microwaves can easily penetrate human skin to a depth of at least a few millimeters. In the considered frequency band, it is also worth mentioning the frequency of 2.16 GHz, at which the average sensitivity of MTM9 reaches its maximum 6.68° per mmol/L, which is more than thirteen times higher than MSTL, with a maximum value of 0.48° per mmol/L.

### 4.4. Numerical Simulations Assessment

Numerical simulation is generally considered a useful tool in the design and testing of electronic components, which offers detailed information about the feasibility of future prototypes in various simulated environments. In the prototype-testing phase, we can modify the general numerical simulation to best reflect actual values measured by the sensor prototype. Validated numerical models can be used for future modifications of sensor geometry or operating frequency. In this work, we used numerical simulations for MSTL and MTM9. We tested the robustness of the computational mesh, the effect of changes in the dielectric properties of the substrate and cover laminate, and the effect of changes in dielectric properties of the liquid phantom in the range of type c uncertainty. After a thorough RMSE analysis of the measured waveform against numerical simulation, we obtained the best correspondence for the amplitude and phase values of *S*_11_ and *S*_21_ parameters for the transition boundary condition while maintaining the initial values of all dielectric parameters. Another interesting phenomenon gathered from numerical simulation and proven by the conducted measurement is the dependence of the resonant frequency *S*_11_ of the MSTL sensor parameter on the change of the dielectric properties of the phantom.

## 5. Conclusions

The proposed metamaterial sensors have demonstrated higher sensitivity of $∠S_{21}$ than standard microstrip transmission line (MSTL) sensors in measuring dielectric parameter changes of blood phantoms over a range of concentrations from 0 to 14 mmol/L. Experiments show that the sensitivity of metamaterial (MTM) sensors increases with the number of cells. For a nine-cell metamaterial sensor (MTM9) at a frequency of 1.99 GHz, the average $∠S_{21}$ sensitivity was determined to be 3.66° per mmol/L, which is significantly higher than the MSTL sensor, with sensitivity of only 0.48° per mmol/L. This effort has proven the feasibility of increasingly sensitive blood glucose sensors based on MTM multicell technology. Future efforts will investigate MTM designs with an increasing number of cells and move on to evaluation of MTM sensors in human blood samples, and eventually in human tissues.

## Figures and Tables

**Table 1:** Calculated values of dielectric properties of liquid phantoms at 2 GHz and room temperature, according to [34].

Sample ID	c_bg_ (mmol/L)	Relative Permittivity	Electrical Conductivity (S/m)
1	0	63.67	1.90
2	3	61.24	1.87
3	6	58.81	1.83
4	10	55.56	1.79
5	14	52.32	1.74

**Table 2:** Dielectric parameters of individual domains of the computational area and the maximum recommended and used edge length of the discretization elements.

Domain	Relative Permittivity *ε_r_*	Electrical Conductivity *σ_e_* (S/m)	Maximum Edge Length of Discretization Elements (mm)
Air	1.00	0.00	3.8
Rogers Ro4003c [38]	3.38	0.8 × 10^−3^	2.0
MT40 [39]	3.45	0.0012	0.9 (*z* axis 0.05)
PLA [40]	2.75	0.00	2.3
Copper MS, MTM	1.00	5.99 × 10^7^	0.35, 0.02
Phantom	Table 5	Table 5	0.45

**Table 3:** Coefficients of relative permittivity and electrical conductivity functions.

	Relative Permittivity—Coefficients of Cubic Functions *p*_0_, *p*_1_, *p*_2_, *p*_3_	Electrical Conductivity—Coefficients of Linear Functions *q*_0_, *q*_1_
Sample ID 1	55.87, 2.08 × 10^−8^, −1.22 × 10^−17^, 2.07 × 10^−27^	−0.71, 1.32 × 10^−9^
Sample ID 2	34.03, 5.10 × 10^−8^, −2.76 × 10^−17^, 4.59 × 10^−27^	−0.97, 1.41 × 10^−9^
Sample ID 3	22.46, 6.60 × 10^−8^, −3.51 × 10^−17^, 5.77 × 10^−27^	−1.16, 1.47 × 10^−9^
Sample ID 4	34.17, 4.41 × 10^−8^, −2.44 × 10^−17^, 4.02 × 10^−27^	−1.24, 1.49 × 10^−9^
Sample ID 5	32.44, 4.17 × 10^−8^, −2.36 × 10^−17^, 3.93 × 10^−27^	−1.29, 1.48 × 10^−9^

**Table 4:** List of phantom ingredients.

Sample ID	DI Water (mL)	IPA (mL)	Sodium Chloride (g)
1	410	90	2.2
2	385	115	1.0
3	370	130	0.6
4	350	150	0.3
5	325	175	0.0

**Table 5:** Measured values of DAK at 2 GHz.

Samle ID	c_bg_ (mmol/L)	Relative Permittivity	Electrical Conductivity (S/m)
1	0	65.0 ± 1.2	1.91 ± 0.05
2	3	62.5 ± 1.2	1.84 ± 0.05
3	6	60.5 ± 1.1	1.77 ± 0.06
4	10	57.2 ± 1.1	1.74 ± 0.04
5	14	53.0 ± 1.0	1.66 ± 0.06

**Table 6:** Average sensor sensitivity at frequency 1.99 GHz for simulation and measurement.

	Sim. Avg. Sens. (° per mmol/L)	Meas. Avg. Sens. (° per mmol/L)
MSTL	0.60	0.48
MTM5	-	1.19
MTM7	-	2.54
MTM9	3.43	3.66
